# The Mitochondrial Response to DNA Damage

**DOI:** 10.3389/fcell.2021.669379

**Published:** 2021-05-12

**Authors:** Ziye Rong, Peipei Tu, Peiqi Xu, Yan Sun, Fangfang Yu, Na Tu, Lixia Guo, Yanan Yang

**Affiliations:** ^1^Department of Immunology, School of Basic Medical Science, Anhui Medical University, Hefei, China; ^2^Department of Microbiology and Bioengineering, School of Life Sciences, Anhui Medical University, Hefei, China; ^3^Division of Pulmonary and Critical Care Medicine, Mayo Clinic, Rochester, MN, United States

**Keywords:** mitochondrial DNA, DNA repair, mitochondrial fusion, mitochondrial fission, mitophagy

## Abstract

Mitochondria are double membrane organelles in eukaryotic cells that provide energy by generating adenosine triphosphate (ATP) through oxidative phosphorylation. They are crucial to many aspects of cellular metabolism. Mitochondria contain their own DNA that encodes for essential proteins involved in the execution of normal mitochondrial functions. Compared with nuclear DNA, the mitochondrial DNA (mtDNA) is more prone to be affected by DNA damaging agents, and accumulated DNA damages may cause mitochondrial dysfunction and drive the pathogenesis of a variety of human diseases, including neurodegenerative disorders and cancer. Therefore, understanding better how mtDNA damages are repaired will facilitate developing therapeutic strategies. In this review, we focus on our current understanding of the mtDNA repair system. We also discuss other mitochondrial events promoted by excessive DNA damages and inefficient DNA repair, such as mitochondrial fusion, fission, and mitophagy, which serve as quality control events for clearing damaged mtDNA.

## Introduction

The mitochondria provide most of the cell energy in the form of adenosine triphosphate (ATP) through oxidative phosphorylation (OXPHOS), which is executed by the electron transport chain (ETC) within the mitochondria. In addition, mitochondria provide other key metabolic intermediates to many other basic cellular processes, such as lipid biogenesis and the synthesis of amino acids (Zong et al., 2016).

In the 1960s, nucleic acids and DNA were discovered in the mitochondria (Saki and Prakash, 2017). The mitochondrial DNA (mtDNA) is circular, and its length is varied in different species, from 75∼85 kb in *Saccharomyces cerevisiae* to around 16.5 kb in mammalian cells (Anderson et al., 1981; Foury et al., 1998). Although most of the mitochondrial proteins are transcribed in the nucleus, synthesized in the cytosol, and then transported into the mitochondria (Gray, 2012), the mtDNA is not non-sense. For example, in human cells, it encodes for 22 tRNAs and two ribosomal RNAs, as well as 13 polypeptides that comprise core subunits of the ETC Complex I, III, IV, and V, which are essential for the OXPHOS activity.

In mammalian cells, mtDNA is tightly linked with proteins to form complexes called nucleoids, which localize to the mitochondrial inner membrane (Chen and Butow, 2005; Holt et al., 2007). Some replication- and transcription-related proteins, such as mitochondrial transcription factor A (TFAM), DNA polymerase gamma (POLG), mitochondrial single-strand binding protein (mtSSB), and mtDNA helicase Twinkle, have been reported to interact with mtDNA (Garrido et al., 2003; Wang and Bogenhagen, 2006; Farge and Falkenberg, 2019).

Similar to nuclear DNA, the mtDNA is constantly assaulted by both exogenous and endogenous stresses, such as ultraviolet (UV) radiation and reactive oxygen species (ROS) (Alexeyev et al., 2013). However, evidence suggests that the mtDNA is more susceptible to certain stress-induced damages (e.g., oxidized DNA damages) than nuclear DNA due to its proximity to the sites of oxidative phosphorylation and lack of the protection by histones (Yakes and Van Houten, 1997; Druzhyna et al., 2008).

Excessive mtDNA damages, if not repaired efficiently, may increase ROS production, which in turn leads to mitochondrial dysfunction and provokes the pathogenesis of many human diseases, including Kearns-Sayre Syndrome, Alzheimer’s, Parkinson’s disease (PD), cancer, and diabetes (Wallace, 2005; Nakabeppu et al., 2007; Tsang et al., 2018; Llanos-Gonzalez et al., 2020). Therefore, accurate maintenance of the mtDNA is essential for a healthy life. In this review, we discuss how mitochondria respond to DNA damages, with an emphasis on how such damages are repaired. We also discuss a series of mitochondrial quality control events, including fission, fusion, and mitophagy, which are important for clearing damaged mtDNA that cannot be efficiently repaired.

## An Overview of the mtDNA Damage Repair

Compared with nuclear DNA repair mechanisms, which have been extensively studied, the mtDNA repair mechanisms are much less understood. Unlike nuclear DNA, mtDNA is multi-copied (Yasukawa and Kang, 2018). Due to such nature, it was previously thought that DNA repair mechanisms might not be present or necessary in the mitochondria. For many years, this had led to a hypothesis that damaged mtDNA was simply degraded without being repaired, while the remained undamaged mtDNA served as the template for mtDNA synthesis (Druzhyna et al., 2008). This hypothesis was primarily based on the experiments showing that pyrimidine dimers induced by UV were not repaired in mammalian mitochondria (Clayton et al., 1974). Given that the nucleotide base repair (NER) is a highly efficient nuclear repair pathway for correcting a variety of DNA damages caused by UV radiation and many other environmental insults (Lee and Kang, 2019), these data suggest that the NER is absent for mtDNA damage repair.

**FIGURE 1:**
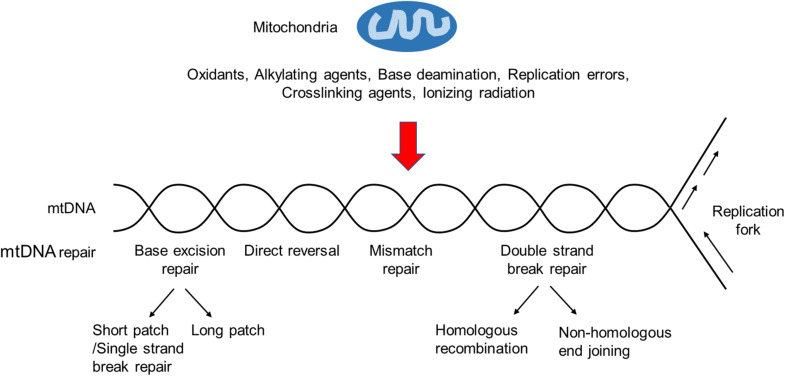
An overview of the mtDNA damage repair. Ionizing radiation, base deamination, replication errors, and DNA damaging agents, such as oxidants, alkylating agents, and crosslinking agents, can cause mtDNA damages. BER is the primary mtDNA damage repair pathway, whereas NER is not observed in mitochondria. In addition, other repair pathways, such as DR, MMR and DSBR, may exist in the mitochondria.

However, more and more studies have shown that several other DNA repair pathways, including base excision repair (BER), direct reversal (DR), mismatch repair (MMR), and possibly double-strand break repair (DSBR), exist in the mammalian mitochondria (Kazak et al., 2012; Saki and Prakash, 2017; Figure 1). Key components of these pathways are encoded by nuclear genes and transported to the mitochondria after being synthesized in the cytosol.

## Base Excision Repair in the Mitochondria

Base excision repair is highly conserved from bacteria to human and has been considered as the primary repair pathway in the mitochondria, responsible for the removal of non-bulky DNA lesions caused predominantly by oxidation, alkylation, deamination, and methylation (Kazak et al., 2012; Krokan and Bjoras, 2013). Its basic process includes damaged DNA base recognition and removal, leaving an apurinic/apyrimidinc (AP) site (or called an abasic site), and subsequently the cleavage of the resulted AP site, end processing, gap filling, and ligation to complete the BER (Fortini et al., 2003).

At the initiation step of the BER, DNA glycosylases play important roles in damaged DNA recognition and removal (Jacobs and Schar, 2012). Seven of eleven known DNA glycosylases have mitochondrial targeting signal and have been identified in mammalian mitochondria (Sharma et al., 2019). They are categorized into two groups, namely the mono-functional glycosylases and bi-functional DNA glycosylases (Mullins et al., 2019). Mono-functional glycosylases include alkyladenine DNA glycosylase (AAG) (van Loon and Samson, 2013), MutY glycosylase homolog (MUTYH) (Ohtsubo et al., 2000), and uracil N-glycosyalse (UNG) (Slupphaug et al., 1993). Bi-functional DNA glycosylases include 8-oxoguanine DNA glycosylase-1 (OGG1) (Nishioka et al., 1999), Nei-like 1 (NEIL1) and Nei-like 2 (NEIL2) (Han et al., 2019), and Nth-Like 1 (NTHL1) (Imai et al., 1998).

Mono-functional glycosylases lack lyase activity and cleave the *N*-glycosidic bonds to generate AP sites, which are further processed by apurinic/apyrimidinic endonuclease 1 (APE1). Bi-functional DNA glycosylases with intrinsic lyase activity are capable of cleaving the abasic sites, and the DNA strand on the 3′ end of the AP site (Kazak et al., 2012; Stein and Sia, 2017). During the end processing, APE1 processes the ends generated by OGG1 and NTHL1 glycosylases (Hegde et al., 2008). The polynucleotide kinase/phosphatase (PNKP) processes the ends generated by the NEIL glycosylases (Tahbaz et al., 2012).

During short-patch BER (SP-BER), 5′-deoxyribose phosphate moiety created by APEI is excised by polymerase γ (Pol γ) with its lyase activity (Longley et al., 1998). Next, Pol γ inserts a single nucleotide to fill the gap. In long patch BER (LP-BER), 2–6 nucleotides are incorporated at the nick by Pol γ through its strand displacement synthesis activity (Longley et al., 1998; Akbari et al., 2008). Then, the 5′ flap is possibly cleaved by flap endonuclease 1 (FEN1) (Liu et al., 2008). Previous reports have shown that other enzymes, including DNA replication helicase/nuclease 2 (DNA2) and 5′-exo/endonuclease (EXOG) are important for removing 5′ flaps during mitochondrial LP-BER (Duxin et al., 2009; Tann et al., 2011). In both SP-BER and LP-BER, DNA ligase III seals the nick to complete the final ligation step of the mitochondrial BER (Simsek et al., 2011).

The single strand break repair (SSBR) pathway is also likely to be present in the mitochondria (Hegde et al., 2012). SSBR can be considered as a form of BER, because many of the proteins involved in BER are important for SSBR. The two pathways have common gap filling and ligation steps. Previously, poly-ADP-ribose polymerase 1 (PARP1), which is required for recognizing single-strand breaks, has been reported to localize to the mitochondria (Rossi et al., 2009). However, the exact role for PARP1 in mammalian mtDNA SSBR is still unclear, and conflicting results have been reported (Szczesny et al., 2014).

During the end processing step, besides of APE1 and PNKP, tyrosyl DNA phosphodiesterase 1 (TDP1) and aprataxin (APTX) have been found to be involved in the mitochondrial SSBR pathway (Hegde et al., 2012; Meagher and Lightowlers, 2014). In the mitochondria, TDP1 typically hydrolyzes the phosphodiester bond between a tyrosyl moiety and a 3′-DNA end. It can also hydrolyze other 3′-end DNA alterations including 3′-phosphoglycolate and 3′-abasic sites. Therefore, TDP1 may function as a general 3′-end-processing DNA repair enzyme to remove a variety of adducts that are poor substrates for APE1 (Das et al., 2010). APTX belongs to the histidine triad (HIT) superfamily of nucleotide hydrolases and transferasese (Kijas et al., 2006). It removes 5′-adenylate (5′-AMP) group from DNA (Zheng et al., 2019). 5′-AMP group is an abortive DNA ligation product during DNA repair and replication, and it must be removed for the later DNA ligation. Previously, a slow rate of 5′-AMP removal was reported in the mitochondrial extracts compared with nuclear extracts (Akbari et al., 2015). Biochemical and cell biological analysis indicated it due to the lack of back up enzymes or repair mechanisms in mtDNA (Caglayan et al., 2017). All these results suggest that APTX is more critical in mtDNA repair than in the nuclear DNA repair.

Notably, mtDNA modifications are also involved in BER. For example, an enrichment of *N*^6^-methyldeoxyadenosine (m^6^A) in mammalian mtDNA has been recently reported (Hao et al., 2020). M^6^A could repress DNA binding and bending by TFAM, which possesses a greater affinity for oxidized lesions and inhibits the activity of OGG1, UNG, and APE1 (Canugovi et al., 2010). Increased m^6^A in the mtDNA affect the binding between TFAM and DNA so that the BER glycosylases are stimulated.

## Other mtDNA Damage Repair Mechanisms

### Direct Reversal

Direct reversal is responsible for the repair of DNA O-alkylated and N-alkylated damages caused by DNA alkylating agents (Fu et al., 2012). This repair process does not require excision, synthesis, and ligation. In mammalian cells, DR is performed by *O*^6^-methylguanine DNA methyltransferase (MGMT) or ALKBH (AlkB homolog) proteins in the nucleus (Ragg et al., 2000; Ahmad et al., 2015). MGMT repairs the *O*^6^-methylguanine lesions by removing methyl groups, whereas ALKBH repairs certain N-alkyl lesions *via* dealkylation. According to previous studies, mitochondrial localization of ALKBH has not been confirmed, and a protein with similar molecular weight to MGMT may localize to mammalian mitochondria (Myers et al., 1988). However, different groups have reported conflicting results, and whether DR is present in the mammalian mitochondria remain to be further clarified (Cai et al., 2005).

### Mismatch Repair

In 2003, MMR activity in rat liver mitochondrial extracts was reported (Mason et al., 2003). Notably, unlike nuclear MMR, there is no evidence that such activity relies on any well-known nuclear MMR proteins, such as MSH and MLH. Instead, it may depend on the Y-box binding protein YB-1 (de Souza-Pinto et al., 2009; Lyabin et al., 2014). This is supported by the facts that depleting YB-1 leads to both a decrease of MMR activity in the mitochondrial extracts and an increase of mtDNA mutations in YB-1-depleted cells. While these findings strongly suggest the presence of YB-1-dependent MMR in the mitochondria, more investigations are needed for better characterizing its detailed mechanism, especially the mitochondrial YB-1-associated cofactor(s) involved in this process.

### Double-Strand Break Repair

Double-strand breaks (DSBs) are the most cytotoxic DNA lesions if not efficiently repaired (Stein and Sia, 2017). DSBs can be induced by ROS, replication stalling, or radiation. In the nucleus, DSBs are mainly repaired by two pathways, including homologous recombination (HR) and non-homologous end joining (NHEJ) pathways (Pannunzio et al., 2018; Wright et al., 2018). HR is considered as an “error-free” repair pathway due to its use of homologous partner as the template to repair DNA lesions. Several lines of evidence support the presence of HR in the mitochondria. For instance, the recombination intermediates containing four-way junctions can be detected in human mitochondria (Pohjoismaki et al., 2009), and Rad51, a RecA homolog, has been reported to execute HR in mammalian mitochondria (Sage et al., 2010). In addition, Rad51 related proteins, e.g., XRCC3, also exist in human mitochondria and may participate in HR (Mishra et al., 2018).

Compared with HR, NHEJ is more error-prone and results in large deletions. DNA end-joining activity has been observed in mitochondrial extracts prepared from mammalian cells, where both cohesive and blunt-ended DNA molecules can be repaired (Lakshmipathy and Campbell, 1999). Breast cancer type 1 susceptibility protein (BRCA1) and p53-binding protein 1 (53BP1) oppositely affect the extent of DNA end resection and are key determinants for the choice of HR or NHEJ (Panier and Boulton, 2014). During G2/S phase, BRCA1 promotes DNA end resection and thus induces HR. During G1 phase, 53BP1 protects the broken DNA end and inhibits long-range end resection, thereby promoting NHEJ. Both BRCA1 and 53BP1 have been identified in the mammalian mitochondria (Coene et al., 2005; Youn et al., 2017).

Taken together, previous findings suggest that both DSB and its related proteins are present in the mitochondria (Coffey and Campbell, 2000; Coene et al., 2005; Sage et al., 2010). However, whether DSB plays a major role in the mtDNA damage repair and whether the mitochondrial HR and NHEJ exert pathophysiological functions in disease contexts remain to be further elucidated.

## The Mitochondrial Response to Unrepaired DNA Damages

**FIGURE 2:**
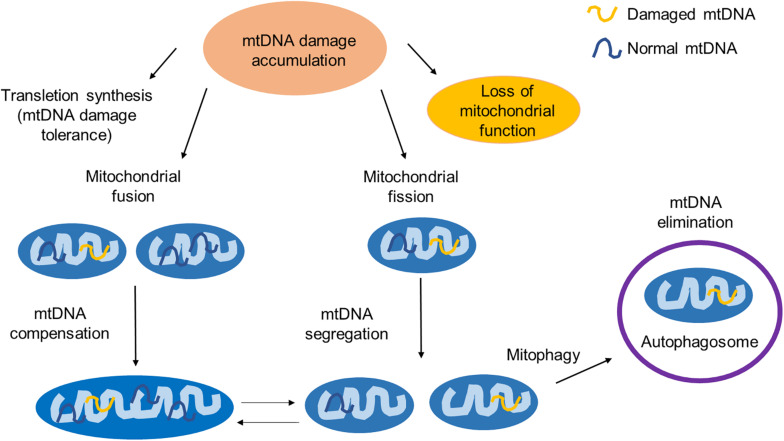
The mitochondrial response to unrepaired DNA damages. Unrepaired mtDNA damages can be accumulated and lead to the mitochondrial dysfunction. To maintain its function, the mitochondria have developed several quality control events. TLS is an mtDNA damage tolerance pathway to bypass the mtDNA replication block. Mitochondrial fusion provides mtDNA complementation by mixing damaged and normal mtDNA. Mitochondrial fission segregates the part of a mitochondrion with damaged mtDNA and maintains the integrity of the healthy part of a mitochondrion. Mitochondria with excessive damaged mtDNA are selectively degraded through mitophagy. The interaction between the mitophagy and the mitochondrial fission/fusion has also been reported.

To maintain a healthy mitochondrial network, mammalian cells have developed several mechanisms to respond to unrepaired DNA damages, including *trans-*lesion synthesis, mitochondrial fusion and fission, and mitophagy. The interactions between some of them have also been reported (Figure 2).

### Mitochondrial DNA Damage Tolerance Pathway: *Trans-*Lesion Synthesis

*Trans-*lesion synthesis (TLS) can bypass the DNA replication block caused by DNA damages (Waters et al., 2009; Sale, 2013). It is an error-prone process, in which the polymerases with low fidelity are involved (Ma X. et al., 2020). In TLS, nucleotides are incorporated to repair DNA lesions but convert them to DNA mutations. It requires DNA polymerase selection and switching (Friedberg et al., 2005). A group of specialized DNA polymerases termed TLS polymerases are utilized in this process, which are categorized into several families (Sale, 2013; Ma X. et al., 2020). Rev1, Polκ, Polη, and Polι belong to the best characterized Y-family polymerases, while polymerase zeta (Polζ) is a B-family polymerase (Yang and Gao, 2018). In mammalian mitochondria, there are evidence that REV3, the catalytic subunit of Polζ localizes to the mitochondria (Singh et al., 2015). REV3 associates with POLG and prevents the mtDNA damage. PrimPol is another TLS polymerase that has been detected in the mammalian mitochondria (Garcia-Gomez et al., 2013; Rudd et al., 2014). This enzyme has both DNA primase activity and DNA polymerase activity. It is capable of bypassing DNA lesions such as 8-oxoG (Guilliam et al., 2015).

### Mitochondrial Fusion, Fission, and Mitophagy

If mtDNA damages cannot be repaired, they will accumulate and subsequently evoke mtDNA quality control events, including mitochondrial fusion, fission, and mitophagy (Youle and van der Bliek, 2012).

Mitochondrial fusion and fission are essential for cell metabolic activities as well as re-distribution of mtDNA (Tilokani et al., 2018). Both fusion and fission are mediated by GTPases (Hoppins et al., 2007). Specifically, Mitofusin 1 (Mfn1), Mitofusin 2 (Mfn2), and Optic Atrophy 1 (OPA1) are involved in the regulation of mitochondrial fusion (Delettre et al., 2000; Chen et al., 2003; Olichon et al., 2003; Detmer and Chan, 2007), and Drp1 is involved in mitochondrial fission (Fonseca et al., 2019).

Damaged and undamaged mtDNAs yield a heteroplasmic mixture of normal and mutant mitochondrial genomes within the same cell (Wonnapinij et al., 2008; Aryaman et al., 2018). Mitochondrial fusion allows the mitochondria with normal mtDNA to compensate for defects in the mitochondria with damaged mtDNA (Nakada et al., 2001; Ono et al., 2001; Yang et al., 2015). Through fusion, essential functional and structural components (e.g., proteins and lipids) in the two types of mitochondria can be diffused and shared, thereby rescuing the mitochondria with damaged mtDNA and mitigating the effects of environmental stresses on the mitochondria if the stress level is below a critical threshold (Stewart and Chinnery, 2015).

Mitochondrial fission allows mtDNAs to be divided into two mitochondria and provides a chance for a mitochondrion to re-fuse with another mitochondrion (Tilokani et al., 2018). It is suggested that fission divides a mitochondrion into two parts and distributes the damaged mtDNA and other harmful components asymmetrically to eliminate the part with most seriously damaged mtDNA but preserve the healthier part with normal mtDNA (Meyer et al., 2017).

Homoplasmy in mtDNA is rarely observed because of inherited or sporadic mutations that result in heteroplasmy (Wei et al., 2020). A broad range of heteroplasmy among different cell types and tissues are observed. The dynamics of heteroplasmy is one of the most challenging aspects of mtDNA disease. The mechanisms regulating heteroplasmy shifts are still needed to be elucidated. In T cell lineage, purifying selection occurs and heteroplasmy ratio is dramatically reduced (Walker et al., 2020). This process may enrich for certain mtDNA sequences and eliminate the pathogenic mutant mtDNA.

Irreparably damaged mtDNA may trigger mitophagy, a process through which depolarized or damaged mitochondria are selectively degraded by autophagy (Lemasters, 2005; Pickles et al., 2018). During autophagy, unwanted cellular materials are sequestered by double-membrane autophagosomes and delivered to lysosomes for degradation (Wollert, 2019). In normal cells, autophagy is utilized to clear and recycle almost all types of cellular materials, including proteins, lipids, damaged organelles, and etc. Mitophagy is an important mitochondrial quality control mechanism to maintain a healthy mitochondrial network (Ma K. et al., 2020). In mammalian cells, two pathways of mitophagy have been described. The first is an ubiquitin-mediated pathway, in which PINK1 (PTEN-induced putative protein kinase 1) has been shown to play an important role by recruiting the E3 ubiquitin ligase Parkin from the cytosol to the mitochondria with damaged DNA (Narendra et al., 2008; Matsuda et al., 2010; Pickrell and Youle, 2015). The other pathway is a receptor-mediated pathway, in which mitophagy receptors on the mitochondria with at least one LC3-interacting region have been identified. Some examples are BCL2 interacting protein 3 (BNIP3) (Chourasia and Macleod, 2015), FUN14 domain containing 1 (FUNDC1) (Chen M. et al., 2016), and mitochondria inner membrane protein Prohibitin 2 (PHB2) (Wei et al., 2017). These receptors mediate mitophagy by binding with LC3 to recruit the autophagosome (Yoo and Jung, 2018).

Previous reports also suggest that mitophagy may be linked to mitochondrial fission and fusion (Shirihai et al., 2015; Yoo and Jung, 2018). For example, overexpression of Opa1 or dominant-negative mutant of Drp1 can inhibit mitophagy (Twig et al., 2008). An interaction between the PINK1/Parkin pathway and the fission/fusion machinery has also been reported (Yu et al., 2011; Murata et al., 2020). It is proposed that fission is prerequisite for mitophagy by segregating the damaged part of a mitochondrion and targeting it for autophagic degradation. It is suspected that the damaged mtDNA and unwanted debris should distribute unevenly in the mitochondria to achieve such asymmetric fission (Zorov et al., 2017). However, the underlying mechanism remains largely unknown.

As above-discussed, the UV-radiation induced mtDNA damages cannot be repaired by mtDNA repair mechanisms. However, the mitochondria with photo-damaged mtDNA undergoes mitophagy (Kim and Lemasters, 2011). It was confirmed that mitophagy can be induced by different types of mtDNA damage stimuli, and it at least partially depends on DNA damage response pathways (Furda et al., 2012). It should be noted that the modulation of mitophagy after DNA damage is independent of the type of mtDNA damage stimuli. Recently, it has been suggested that Spata18, a p53 inducible protein, is critical in mitophagy after mtDNA damage (Dan et al., 2020). Knocking down Spata18 suppresses mitophagy, promotes mtDNA damage, and attenuates mtDNA repair. To better understand the mitophagy process in response to mtDNA damage, it is important to identify related proteins and figure out how a mitochondrion, or its part, with damaged mtDNA can be selectively removed.

## Mitophagy in Immunity and Human Diseases

The interplay of mitophagy with mitochondrial dynamics is critical for maintaining mitochondrial homeostasis in normal and stressed conditions. Damaged mitochondria may either induce innate immunity or trigger cell death to drive aging-related diseases when mtDNA damages are beyond repairable (Ma K. et al., 2020).

Previous reports suggest that the mtDNA is implicated in the regulation of inflammation and innate immunity, and a major mediator for these processes is the “cGAS-STING” pathway (West et al., 2015; Chen Q. et al., 2016). For instance, internalized bacterial endotoxin lipopolysaccharide (LPS) can trjgger the mitochondrial membrane localization of active GSDMD, which in turn stimulates the release of mtDNA into the cytosol (Huang et al., 2020). The released mtDNA can be detected by the cyclic AMP-GMP synthase (cGAS), which generates cyclic GMP-AMP (cGAMP) to activate the pro-inflammatory stimulator of interferon genes (STING), eventually leading to increased production of type 1 interferons (IFNs).

In cancer cells, the mitochondrial outer membrane permeabilization (MOMP) has been shown to promote the cytosolic release of mtDNA, the activation of the cGAS-STING pathway, and the production of type I IFNs, which collectively facilitate an effective radiation therapy (Yamazaki et al., 2020); whereas Bcl2-dependent mitophagy can deplete MOMP, thereby limiting the release of mtDNA and associated immune responses.

Age-related accumulation of mtDNA mutations in human somatic and germ cells have also been reported (Arbeithuber et al., 2020). Replication errors are likely the main sources of *de novo* mtDNA mutations. Accumulation of certain types of mtDNA mutations may trigger cell death and lead to age-related diseases, such as PD, Cockayne syndrome (CS), and Werner syndrome (WS) (Palikaras et al., 2015; Fang et al., 2019; Lopes et al., 2020).

Parkinson’s disease is a progressive neurodegenerative disorder with movement problems due to the loss of dopamine-producing neurons (Kalia and Lang, 2015). Interestingly, most of PD-related proteins have been shown to lead to dysfunctional mitochondria and/or accumulated damaged mitochondria (Martin-Jimenez et al., 2020). Some of them, including PINK1 and Parkin and LRRK2, play important roles in the execution of mitophagy (Vives-Bauza et al., 2010; Wallings et al., 2015). These facts not only establish a strong link between mitophagy and the pathogenesis of PD, but also suggest that manipulating mitophagy may have diagnostic/therapeutic values.

Werner syndrome is a human premature aging disease caused by mutations in the gene encoding for Werner protein (WRN), which is an important DNA helicase involved in DNA repair (Fang et al., 2019). WRN regulates the transcription of nicotinamide nucleotide adenylyltransferase 1 and NAD^+^ biosynthesis. Accordingly, decreased NAD^+^ levels and impaired mitophagy have been reported in WS patient samples.

In CS patient samples, there is a reduction in the activation of AMP-activated kinase (AMPK) and its downstream target, autophagy protein unc-51 like autophagy activating kinase 1 (ULK1), suggesting dysfunctional mitochondrial dynamics and impaired mitophagy (Okur et al., 2020). Reduced NAD^+^ metabolism level was discovered in all three diseases. NAD^+^ precursors can be used for treatment by promoting mitophagy *via* increasing the levels of key mitophagy-related proteins, such as PINK1, ULK1, and DCT-1 (Aman et al., 2020). Mitochondrial quality is improved and mitochondrial defects are reversed. These results further implicate that promoting mitophagy can be a promising approach to treat some age-related diseases.

## Conclusion

We have known that cells are continuously exposed to both internal and external stresses, which may cause mtDNA damages. In response to such damages, the mitochondria have developed multiple ways to maintain its DNA integrity and function. Nevertheless, we still lack a comprehensive understanding of how distinct pathways/mechanisms act together to maintain a functional mitochondrial genome.

In this review, we have mainly focused on the mitochondrial response that repairs, attenuates, or eliminate mtDNA damages. Compared with nuclear DNA repair pathways, the pathways for repairing mtDNA damages are less understood. Many nuclear DNA repair proteins, especially those found in the nuclear BER pathway, are also found in the mitochondria (Supplementary Table 1). Previous data suggest that BER is the major mtDNA repair pathway, whereas NER has not been confirmed in the mitochondria. DR, MMR and DSBR may exist in the mitochondria. However, key components involved in these pathways have not been fully elucidated. To that end, further efforts are needed to identify the proteins/enzymes participating in distinct mtDNA damage repair processes.

Mitochondria have multiple quality control pathways to maintain its function in response to stresses. Herein, we have briefly discussed several such pathways, including TLS, mitochondrial fission, fusion, and mitophagy. TLS can also be considered as a post-replication repair pathway. It utilizes damaged mtDNA as template to replicate with low-fidelity polymerases. Mitochondrial fusion and fission are dynamic to balance mtDNA damage compensation and elimination, and mitophagy is primarily responsible for the elimination of mitochondria with damaged mtDNA. Mis-regulation of such mtDNA damage responses has been linked to the immunity and pathogenesis of human diseases, including neurodegenerative disorders and cancer. In-depth understanding of these processes will provide insights for developing novel treatment strategies.

## Author Contributions

ZR and YY wrote the manuscript. All authors discussed and approved the contents.

## Conflict of Interest

The authors declare that the research was conducted in the absence of any commercial or financial relationships that could be construed as a potential conflict of interest.

## Supplementary Material

Supplementary Table 1 Enzymes/proteins in mtDNA repair. Important enzymes/proteins in mammalian mitochondria in different mtDNA damage repair mechanisms are present. Functions of each enzyme/protein are also listed.

## References

[B1] Ahmad A. Nay S. L. O’Connor T. R. (2015). Direct reversal repair in mammalian cells. *Adv. DNA Repair* 95–128. 10.5772/60037

[B2] Akbari M. Sykora P. Bohr V. A. (2015). Slow mitochondrial repair of 5’-AMP renders mtDNA susceptible to damage in APTX deficient cells. *Sci. Rep.* 5:12876. 10.1038/srep12876 26256098 PMC4530458

[B3] Akbari M. Visnes T. Krokan H. E. Otterlei M. (2008). Mitochondrial base excision repair of uracil and AP sites takes place by single-nucleotide insertion and long-patch DNA synthesis. *DNA Repair (Amst.)* 7 605–616. 10.1016/j.dnarep.2008.01.002 18295553

[B4] Alexeyev M. Shokolenko I. Wilson G. LeDoux S. (2013). The maintenance of mitochondrial DNA integrity–critical analysis and update. *Cold Spring Harb. Perspect. Biol.* 5:a012641. 10.1101/cshperspect.a012641 23637283 PMC3632056

[B5] Aman Y. Frank J. Lautrup S. H. Matysek A. Niu Z. Yang G. (2020). The NAD(+)-mitophagy axis in healthy longevity and in artificial intelligence-based clinical applications. *Mech. Ageing Dev.* 185:111194. 10.1016/j.mad.2019.111194 31812486 PMC7545219

[B6] Anderson S. Bankier A. T. Barrell B. G. de Bruijn M. H. Coulson A. R. Drouin J. (1981). Sequence and organization of the human mitochondrial genome. *Nature* 290 457–465. 10.1038/290457a0 7219534

[B7] Arbeithuber B. Hester J. Cremona M. A. Stoler N. Zaidi A. Higgins B. (2020). Age-related accumulation of de novo mitochondrial mutations in mammalian oocytes and somatic tissues. *PLoS Biol.* 18:e3000745. 10.1371/journal.pbio.3000745 32667908 PMC7363077

[B8] Aryaman J. Johnston I. G. Jones N. S. (2018). Mitochondrial heterogeneity. *Front. Genet.* 9:718. 10.3389/fgene.2018.00718 30740126 PMC6355694

[B9] Caglayan M. Prasad R. Krasich R. Longley M. J. Kadoda K. Tsuda M. (2017). Complementation of aprataxin deficiency by base excision repair enzymes in mitochondrial extracts. *Nucleic Acids Res.* 45 10079–10088. 10.1093/nar/gkx654 28973450 PMC5622373

[B10] Cai S. Xu Y. Cooper R. J. Ferkowicz M. J. Hartwell J. R. Pollok K. E. (2005). Mitochondrial targeting of human O6-methylguanine DNA methyltransferase protects against cell killing by chemotherapeutic alkylating agents. *Cancer Res.* 65 3319–3327. 10.1158/0008-5472.CAN-04-3335 15833865

[B11] Canugovi C. Maynard S. Bayne A. C. Sykora P. Tian J. de Souza-Pinto N. C. (2010). The mitochondrial transcription factor A functions in mitochondrial base excision repair. *DNA Repair (Amst.)* 9 1080–1089. 10.1016/j.dnarep.2010.07.009 20739229 PMC2955416

[B12] Chen H. Detmer S. A. Ewald A. J. Griffin E. E. Fraser S. E. Chan D. C. (2003). Mitofusins Mfn1 and Mfn2 coordinately regulate mitochondrial fusion and are essential for embryonic development. *J. Cell Biol.* 160 189–200. 10.1083/jcb.200211046 12527753 PMC2172648

[B13] Chen M. Chen Z. Wang Y. Tan Z. Zhu C. Li Y. (2016). Mitophagy receptor FUNDC1 regulates mitochondrial dynamics and mitophagy. *Autophagy* 12 689–702. 10.1080/15548627.2016.1151580 27050458 PMC4836026

[B14] Chen Q. Sun L. Chen Z. J. (2016). Regulation and function of the cGAS-STING pathway of cytosolic DNA sensing. *Nat. Immunol.* 17 1142–1149. 10.1038/ni.3558 27648547

[B15] Chen X. J. Butow R. A. (2005). The organization and inheritance of the mitochondrial genome. *Nat. Rev. Genet.* 6 815–825. 10.1038/nrg1708 16304597

[B16] Chourasia A. H. Macleod K. F. (2015). Tumor suppressor functions of BNIP3 and mitophagy. *Autophagy* 11 1937–1938. 10.1080/15548627.2015.1085136 26315353 PMC4824596

[B17] Clayton D. A. Doda J. N. Friedberg E. C. (1974). The absence of a pyrimidine dimer repair mechanism in mammalian mitochondria. *Proc. Natl. Acad. Sci. U. S. A.* 71 2777–2781. 10.1073/pnas.71.7.2777 4212385 PMC388554

[B18] Coene E. D. Hollinshead M. S. Waeytens A. A. Schelfhout V. R. Eechaute W. P. Shaw M. K. (2005). Phosphorylated BRCA1 is predominantly located in the nucleus and mitochondria. *Mol. Biol. Cell* 16 997–1010. 10.1091/mbc.e04-10-0895 15591126 PMC545929

[B19] Coffey G. Campbell C. (2000). An alternate form of Ku80 is required for DNA end-binding activity in mammalian mitochondria. *Nucleic Acids Res.* 28 3793–3800. 10.1093/nar/28.19.3793 11000272 PMC110772

[B20] Dan X. Babbar M. Moore A. Wechter N. Tian J. Mohanty J. G. (2020). DNA damage invokes mitophagy through a pathway involving Spata18. *Nucleic Acids Res.* 48 6611–6623. 10.1093/nar/gkaa393 32453416 PMC7337932

[B21] Das B. B. Dexheimer T. S. Maddali K. Pommier Y. (2010). Role of tyrosyl-DNA phosphodiesterase (TDP1) in mitochondria. *Proc. Natl. Acad. Sci. U. S. A.* 107 19790–19795. 10.1073/pnas.1009814107 21041670 PMC2993338

[B22] de Souza-Pinto N. C. Mason P. A. Hashiguchi K. Weissman L. Tian J. Guay D. (2009). Novel DNA mismatch-repair activity involving YB-1 in human mitochondria. *DNA Repair (Amst.)* 8 704–719. 10.1016/j.dnarep.2009.01.021 19272840 PMC2693314

[B23] Delettre C. Lenaers G. Griffoin J. M. Gigarel N. Lorenzo C. Belenguer P. (2000). Nuclear gene OPA1, encoding a mitochondrial dynamin-related protein, is mutated in dominant optic atrophy. *Nat. Genet.* 26 207–210. 10.1038/79936 11017079

[B24] Detmer S. A. Chan D. C. (2007). Complementation between mouse Mfn1 and Mfn2 protects mitochondrial fusion defects caused by CMT2A disease mutations. *J. Cell Biol.* 176 405–414. 10.1083/jcb.200611080 17296794 PMC2063976

[B25] Druzhyna N. M. Wilson G. L. LeDoux S. P. (2008). Mitochondrial DNA repair in aging and disease. *Mech. Ageing Dev.* 129 383–390. 10.1016/j.mad.2008.03.002 18417187 PMC2666190

[B26] Duxin J. P. Dao B. Martinsson P. Rajala N. Guittat L. Campbell J. L. (2009). Human Dna2 is a nuclear and mitochondrial DNA maintenance protein. *Mol. Cell Biol.* 29 4274–4282. 10.1128/MCB.01834-08 19487465 PMC2715806

[B27] Fang E. F. Hou Y. Lautrup S. Jensen M. B. Yang B. SenGupta T. (2019). NAD(+) augmentation restores mitophagy and limits accelerated aging in Werner syndrome. *Nat. Commun.* 10:5284. 10.1038/s41467-019-13172-8 31754102 PMC6872719

[B28] Farge G. Falkenberg M. (2019). Organization of DNA in mammalian mitochondria. *Int. J. Mol. Sci.* 20:2770. 10.3390/ijms20112770 31195723 PMC6600607

[B29] Fonseca T. B. Sanchez-Guerrero A. Milosevic I. Raimundo N. (2019). Mitochondrial fission requires DRP1 but not dynamins. *Nature* 570 E34–E42. 10.1038/s41586-019-1296-y 31217603

[B30] Fortini P. Pascucci B. Parlanti E. D’Errico M. Simonelli V. Dogliotti E. (2003). The base excision repair: mechanisms and its relevance for cancer susceptibility. *Biochimie* 85 1053–1071. 10.1016/j.biochi.2003.11.003 14726013

[B31] Foury F. Roganti T. Lecrenier N. Purnelle B. (1998). The complete sequence of the mitochondrial genome of Saccharomyces cerevisiae. *FEBS Lett.* 440 325–331. 10.1016/s0014-5793(98)01467-7 9872396

[B32] Friedberg E. C. Lehmann A. R. Fuchs R. P. (2005). Trading places: how do DNA polymerases switch during translesion DNA synthesis? *Mol. Cell* 18 499–505. 10.1016/j.molcel.2005.03.032 15916957

[B33] Fu D. Calvo J. A. Samson L. D. (2012). Balancing repair and tolerance of DNA damage caused by alkylating agents. *Nat. Rev. Cancer* 12 104–120. 10.1038/nrc3185 22237395 PMC3586545

[B34] Furda A. M. Marrangoni A. M. Lokshin A. Van Houten B. (2012). Oxidants and not alkylating agents induce rapid mtDNA loss and mitochondrial dysfunction. *DNA Repair (Amst.)* 11 684–692. 10.1016/j.dnarep.2012.06.002 22766155 PMC3878289

[B35] Garcia-Gomez S. Reyes A. Martinez-Jimenez M. I. Chocron E. S. Mouron S. Terrados G. (2013). PrimPol, an archaic primase/polymerase operating in human cells. *Mol. Cell* 52 541–553. 10.1016/j.molcel.2013.09.025 24207056 PMC3899013

[B36] Garrido N. Griparic L. Jokitalo E. Wartiovaara J. van der Bliek A. M. Spelbrink J. N. (2003). Composition and dynamics of human mitochondrial nucleoids. *Mol. Biol. Cell* 14 1583–1596. 10.1091/mbc.e02-07-0399 12686611 PMC153124

[B37] Gray M. W. (2012). Mitochondrial evolution. *Cold Spring Harb. Perspect. Biol.* 4:a011403. 10.1101/cshperspect.a011403 22952398 PMC3428767

[B38] Guilliam T. A. Jozwiakowski S. K. Ehlinger A. Barnes R. P. Rudd S. G. Bailey L. J. (2015). Human PrimPol is a highly error-prone polymerase regulated by single-stranded DNA binding proteins. *Nucleic Acids Res.* 43 1056–1068. 10.1093/nar/gku1321 25550423 PMC4333378

[B39] Han D. Schomacher L. Schule K. M. Mallick M. Musheev M. U. Karaulanov E. (2019). NEIL1 and NEIL2 DNA glycosylases protect neural crest development against mitochondrial oxidative stress. *Elife* 8:e49044. 10.7554/eLife.49044 31566562 PMC6768664

[B40] Hao Z. Wu T. Cui X. Zhu P. Tan C. Dou X. (2020). N(6)-deoxyadenosine methylation in mammalian mitochondrial DNA. *Mol. Cell* 78 382–395.e8. 10.1016/j.molcel.2020.02.018 32183942 PMC7214128

[B41] Hegde M. L. Hazra T. K. Mitra S. (2008). Early steps in the DNA base excision/single-strand interruption repair pathway in mammalian cells. *Cell Res.* 18 27–47. 10.1038/cr.2008.8 18166975 PMC2692221

[B42] Hegde M. L. Mantha A. K. Hazra T. K. Bhakat K. K. Mitra S. Szczesny B. (2012). Oxidative genome damage and its repair: implications in aging and neurodegenerative diseases. *Mech. Ageing Dev.* 133 157–168. 10.1016/j.mad.2012.01.005 22313689 PMC3351531

[B43] Holt I. J. He J. Mao C. C. Boyd-Kirkup J. D. Martinsson P. Sembongi H. (2007). Mammalian mitochondrial nucleoids: organizing an independently minded genome. *Mitochondrion* 7 311–321. 10.1016/j.mito.2007.06.004 17698423

[B44] Hoppins S. Lackner L. Nunnari J. (2007). The machines that divide and fuse mitochondria. *Annu. Rev. Biochem.* 76 751–780. 10.1146/annurev.biochem.76.071905.090048 17362197

[B45] Huang L. S. Hong Z. Wu W. Xiong S. Zhong M. Gao X. (2020). mtDNA activates cGAS signaling and suppresses the YAP-mediated endothelial cell proliferation program to promote inflammatory injury. *Immunity* 52 475–486.e5. 10.1016/j.immuni.2020.02.002 32164878 PMC7266657

[B46] Imai K. Sarker A. H. Akiyama K. Ikeda S. Yao M. Tsutsui K. (1998). Genomic structure and sequence of a human homologue (NTHL1/NTH1) of *Escherichia coli* endonuclease III with those of the adjacent parts of TSC2 and SLC9A3R2 genes. *Gene* 222 287–295. 10.1016/s0378-1119(98)00485-5 9831664

[B47] Jacobs A. L. Schar P. (2012). DNA glycosylases: in DNA repair and beyond. *Chromosoma* 121 1–20. 10.1007/s00412-011-0347-4 22048164 PMC3260424

[B48] Kalia L. V. Lang A. E. (2015). Parkinson’s disease. *Lancet* 386 896–912. 10.1016/S0140-6736(14)61393-3 25904081

[B49] Kazak L. Reyes A. Holt I. J. (2012). Minimizing the damage: repair pathways keep mitochondrial DNA intact. *Nat. Rev. Mol. Cell Biol.* 13 659–671. 10.1038/nrm3439 22992591

[B50] Kijas A. W. Harris J. L. Harris J. M. Lavin M. F. (2006). Aprataxin forms a discrete branch in the HIT (histidine triad) superfamily of proteins with both DNA/RNA binding and nucleotide hydrolase activities. *J. Biol. Chem.* 281 13939–13948. 10.1074/jbc.M507946200 16547001

[B51] Kim I. Lemasters J. J. (2011). Mitophagy selectively degrades individual damaged mitochondria after photoirradiation. *Antioxid. Redox Signal.* 14 1919–1928. 10.1089/ars.2010.3768 21126216 PMC3078512

[B52] Krokan H. E. Bjoras M. (2013). Base excision repair. *Cold Spring Harb. Perspect. Biol.* 5:a012583. 10.1101/cshperspect.a012583 23545420 PMC3683898

[B53] Lakshmipathy U. Campbell C. (1999). Double strand break rejoining by mammalian mitochondrial extracts. *Nucleic Acids Res.* 27 1198–1204. 10.1093/nar/27.4.1198 9927756 PMC148303

[B54] Lee T. H. Kang T. H. (2019). DNA oxidation and excision repair pathways. *Int. J. Mol. Sci.* 20:6092. 10.3390/ijms20236092 31816862 PMC6929053

[B55] Lemasters J. J. (2005). Selective mitochondrial autophagy, or mitophagy, as a targeted defense against oxidative stress, mitochondrial dysfunction, and aging. *Rejuvenation Res.* 8 3–5. 10.1089/rej.2005.8.3 15798367

[B56] Liu P. Qian L. Sung J. S. de Souza-Pinto N. C. Zheng L. Bogenhagen D. F. (2008). Removal of oxidative DNA damage via FEN1-dependent long-patch base excision repair in human cell mitochondria. *Mol. Cell Biol.* 28 4975–4987. 10.1128/MCB.00457-08 18541666 PMC2519700

[B57] Llanos-Gonzalez E. Henares-Chavarino A. A. Pedrero-Prieto C. M. Garcia-Carpintero S. Frontinan-Rubio J. Sancho-Bielsa F. J. (2020). Interplay between mitochondrial oxidative disorders and proteostasis in Alzheimer’s disease. *Front. Neurosci.* 13:ARTN1444. 10.3389/fnins.2019.01444 32063825 PMC7000623

[B58] Longley M. J. Prasad R. Srivastava D. K. Wilson S. H. Copeland W. C. (1998). Identification of 5’-deoxyribose phosphate lyase activity in human DNA polymerase gamma and its role in mitochondrial base excision repair in vitro. *Proc. Natl. Acad. Sci. U. S. A.* 95 12244–12248. 10.1073/pnas.95.21.12244 9770471 PMC22816

[B59] Lopes A. F. C. Bozek K. Herholz M. Trifunovic A. Rieckher M. Schumacher B. (2020). A C. elegans model for neurodegeneration in Cockayne syndrome. *Nucleic Acids Res.* 48 10973–10985. 10.1093/nar/gkaa795 33021672 PMC7641758

[B60] Lyabin D. N. Eliseeva I. A. Ovchinnikov L. P. (2014). YB-1 protein: functions and regulation. *Wiley Interdiscip. Rev. RNA* 5 95–110. 10.1002/wrna.1200 24217978

[B61] Ma K. Chen G. Li W. Kepp O. Zhu Y. Chen Q. (2020). Mitophagy, mitochondrial homeostasis, and cell fate. *Front. Cell Dev. Biol.* 8:467. 10.3389/fcell.2020.00467 32671064 PMC7326955

[B62] Ma X. Tang T. S. Guo C. (2020). Regulation of translesion DNA synthesis in mammalian cells. *Environ. Mol. Mutagen.* 61 680–692. 10.1002/em.22359 31983077

[B63] Martin-Jimenez R. Lurette O. Hebert-Chatelain E. (2020). Damage in mitochondrial DNA associated with Parkinson’s disease. *DNA Cell Biol.* 39 1421–1430. 10.1089/dna.2020.5398 32397749

[B64] Mason P. A. Matheson E. C. Hall A. G. Lightowlers R. N. (2003). Mismatch repair activity in mammalian mitochondria. *Nucleic Acids Res.* 31 1052–1058. 10.1093/nar/gkg167 12560503 PMC149189

[B65] Matsuda N. Sato S. Shiba K. Okatsu K. Saisho K. Gautier C. A. (2010). PINK1 stabilized by mitochondrial depolarization recruits Parkin to damaged mitochondria and activates latent Parkin for mitophagy. *J. Cell Biol.* 189 211–221. 10.1083/jcb.200910140 20404107 PMC2856912

[B66] Meagher M. Lightowlers R. N. (2014). The role of TDP1 and APTX in mitochondrial DNA repair. *Biochimie* 100 121–124. 10.1016/j.biochi.2013.10.011 24161509 PMC4356151

[B67] Meyer J. N. Leuthner T. C. Luz A. L. (2017). Mitochondrial fusion, fission, and mitochondrial toxicity. *Toxicology* 391 42–53. 10.1016/j.tox.2017.07.019 28789970 PMC5681418

[B68] Mishra A. Saxena S. Kaushal A. Nagaraju G. (2018). RAD51C/XRCC3 facilitates mitochondrial DNA replication and maintains integrity of the mitochondrial genome. *Mol. Cell Biol.* 38:e00489–17. 10.1128/MCB.00489-17 29158291 PMC5770535

[B69] Mullins E. A. Rodriguez A. A. Bradley N. P. Eichman B. F. (2019). Emerging roles of DNA glycosylases and the base excision repair pathway. *Trends Biochem. Sci.* 44 765–781. 10.1016/j.tibs.2019.04.006 31078398 PMC6699911

[B70] Murata D. Arai K. Iijima M. Sesaki H. (2020). Mitochondrial division, fusion and degradation. *J. Biochem.* 167 233–241. 10.1093/jb/mvz106 31800050 PMC7048076

[B71] Myers K. A. Saffhill R. O’Connor P. J. (1988). Repair of alkylated purines in the hepatic DNA of mitochondria and nuclei in the rat. *Carcinogenesis* 9 285–292. 10.1093/carcin/9.2.285 3338112

[B72] Nakabeppu Y. Tsuchimoto D. Yamaguchi H. Sakumi K. (2007). Oxidative damage in nucleic acids and Parkinson’s disease. *J. Neurosci. Res.* 85 919–934. 10.1002/jnr.21191 17279544

[B73] Nakada K. Inoue K. Ono T. Isobe K. Ogura A. Goto Y. I. (2001). Inter-mitochondrial complementation: mitochondria-specific system preventing mice from expression of disease phenotypes by mutant mtDNA. *Nat. Med.* 7 934–940. 10.1038/90976 11479626

[B74] Narendra D. Tanaka A. Suen D. F. Youle R. J. (2008). Parkin is recruited selectively to impaired mitochondria and promotes their autophagy. *J. Cell Biol.* 183 795–803. 10.1083/jcb.200809125 19029340 PMC2592826

[B75] Nishioka K. Ohtsubo T. Oda H. Fujiwara T. Kang D. Sugimachi K. (1999). Expression and differential intracellular localization of two major forms of human 8-oxoguanine DNA glycosylase encoded by alternatively spliced OGG1 mRNAs. *Mol. Biol. Cell* 10 1637–1652. 10.1091/mbc.10.5.1637 10233168 PMC30487

[B76] Ohtsubo T. Nishioka K. Imaiso Y. Iwai S. Shimokawa H. Oda H. (2000). Identification of human MutY homolog (hMYH) as a repair enzyme for 2-hydroxyadenine in DNA and detection of multiple forms of hMYH located in nuclei and mitochondria. *Nucleic Acids Res.* 28 1355–1364. 10.1093/nar/28.6.1355 10684930 PMC111038

[B77] Okur M. N. Fang E. F. Fivenson E. M. Tiwari V. Croteau D. L. Bohr V. A. (2020). Cockayne syndrome proteins CSA and CSB maintain mitochondrial homeostasis through NAD(+) signaling. *Aging Cell* 19:e13268. 10.1111/acel.13268 33166073 PMC7744955

[B78] Olichon A. Baricault L. Gas N. Guillou E. Valette A. Belenguer P. (2003). Loss of OPA1 perturbates the mitochondrial inner membrane structure and integrity, leading to cytochrome c release and apoptosis. *J. Biol. Chem.* 278 7743–7746. 10.1074/jbc.C200677200 12509422

[B79] Ono T. Isobe K. Nakada K. Hayashi J. I. (2001). Human cells are protected from mitochondrial dysfunction by complementation of DNA products in fused mitochondria. *Nat. Genet.* 28 272–275. 10.1038/90116 11431699

[B80] Palikaras K. Lionaki E. Tavernarakis N. (2015). Coordination of mitophagy and mitochondrial biogenesis during ageing in C. elegans. *Nature* 521 525–528. 10.1038/nature14300 25896323

[B81] Panier S. Boulton S. J. (2014). Double-strand break repair: 53BP1 comes into focus. *Nat. Rev. Mol. Cell Biol.* 15 7–18. 10.1038/nrm3719 24326623

[B82] Pannunzio N. R. Watanabe G. Lieber M. R. (2018). Nonhomologous DNA end-joining for repair of DNA double-strand breaks. *J. Biol. Chem.* 293 10512–10523. 10.1074/jbc.TM117.000374 29247009 PMC6036208

[B83] Pickles S. Vigie P. Youle R. J. (2018). Mitophagy and quality control mechanisms in mitochondrial maintenance. *Curr. Biol.* 28 R170–R185. 10.1016/j.cub.2018.01.004 29462587 PMC7255410

[B84] Pickrell A. M. Youle R. J. (2015). The roles of PINK1, parkin, and mitochondrial fidelity in Parkinson’s disease. *Neuron* 85 257–273. 10.1016/j.neuron.2014.12.007 25611507 PMC4764997

[B85] Pohjoismaki J. L. Goffart S. Tyynismaa H. Willcox S. Ide T. Kang D. (2009). Human heart mitochondrial DNA is organized in complex catenated networks containing abundant four-way junctions and replication forks. *J. Biol. Chem.* 284 21446–21457. 10.1074/jbc.M109.016600 19525233 PMC2755869

[B86] Ragg S. Xu-Welliver M. Bailey J. D’Souza M. Cooper R. Chandra S. (2000). Direct reversal of DNA damage by mutant methyltransferase protein protects mice against dose-intensified chemotherapy and leads to in vivo selection of hematopoietic stem cells. *Cancer Res.* 60 5187–5195. 11016647

[B87] Rossi M. N. Carbone M. Mostocotto C. Mancone C. Tripodi M. Maione R. (2009). Mitochondrial localization of PARP-1 requires interaction with mitofilin and is involved in the maintenance of mitochondrial DNA integrity. *J. Biol. Chem.* 284 31616–31624. 10.1074/jbc.M109.025882 19762472 PMC2797232

[B88] Rudd S. G. Bianchi J. Doherty A. J. (2014). PrimPol-A new polymerase on the block. *Mol. Cell Oncol.* 1:e960754. 10.4161/23723548.2014.960754 27308331 PMC4905188

[B89] Sage J. M. Gildemeister O. S. Knight K. L. (2010). Discovery of a novel function for human Rad51: maintenance of the mitochondrial genome. *J. Biol. Chem.* 285 18984–18990. 10.1074/jbc.M109.099846 20413593 PMC2885175

[B90] Saki M. Prakash A. (2017). DNA damage related crosstalk between the nucleus and mitochondria. *Free Radic. Biol. Med.* 107 216–227. 10.1016/j.freeradbiomed.2016.11.050 27915046 PMC5449269

[B91] Sale J. E. (2013). Translesion DNA synthesis and mutagenesis in eukaryotes. *Cold Spring Harb. Perspect. Biol.* 5:a012708. 10.1101/cshperspect.a012708 23457261 PMC3578355

[B92] Sharma N. Pasala M. S. Prakash A. (2019). Mitochondrial DNA: epigenetics and environment. *Environ. Mol. Mutagen.* 60 668–682. 10.1002/em.22319 31335990 PMC6941438

[B93] Shirihai O. S. Song M. Dorn G. W. II (2015). How mitochondrial dynamism orchestrates mitophagy. *Circ. Res.* 116 1835–1849. 10.1161/CIRCRESAHA.116.306374 25999423 PMC4443843

[B94] Simsek D. Furda A. Gao Y. Artus J. Brunet E. Hadjantonakis A. K. (2011). Crucial role for DNA ligase III in mitochondria but not in Xrcc1-dependent repair. *Nature* 471 245–248. 10.1038/nature09794 21390132 PMC3261757

[B95] Singh B. Li X. Owens K. M. Vanniarajan A. Liang P. Singh K. K. (2015). Human REV3 DNA polymerase zeta localizes to mitochondria and protects the mitochondrial genome. *PLoS One* 10:e0140409. 10.1371/journal.pone.0140409 26462070 PMC4604079

[B96] Slupphaug G. Markussen F. H. Olsen L. C. Aasland R. Aarsaether N. Bakke O. (1993). Nuclear and mitochondrial forms of human uracil-DNA glycosylase are encoded by the same gene. *Nucleic Acids Res.* 21 2579–2584. 10.1093/nar/21.11.2579 8332455 PMC309584

[B97] Stein A. Sia E. A. (2017). Mitochondrial DNA repair and damage tolerance. *Front. Biosci. (Landmark Ed.)* 22:920–943. 10.2741/4525 27814655

[B98] Stewart J. B. Chinnery P. F. (2015). The dynamics of mitochondrial DNA heteroplasmy: implications for human health and disease. *Nat. Rev. Genet.* 16 530–542. 10.1038/nrg3966 26281784

[B99] Szczesny B. Brunyanszki A. Olah G. Mitra S. Szabo C. (2014). Opposing roles of mitochondrial and nuclear PARP1 in the regulation of mitochondrial and nuclear DNA integrity: implications for the regulation of mitochondrial function. *Nucleic Acids Res.* 42 13161–13173. 10.1093/nar/gku1089 25378300 PMC4245951

[B100] Tahbaz N. Subedi S. Weinfeld M. (2012). Role of polynucleotide kinase/phosphatase in mitochondrial DNA repair. *Nucleic Acids Res.* 40 3484–3495. 10.1093/nar/gkr1245 22210862 PMC3333865

[B101] Tann A. W. Boldogh I. Meiss G. Qian W. Van Houten B. Mitra S. (2011). Apoptosis induced by persistent single-strand breaks in mitochondrial genome: critical role of EXOG (5’-EXO/endonuclease) in their repair. *J. Biol. Chem.* 286 31975–31983. 10.1074/jbc.M110.215715 21768646 PMC3173182

[B102] Tilokani L. Nagashima S. Paupe V. Prudent J. (2018). Mitochondrial dynamics: overview of molecular mechanisms. *Essays Biochem.* 62 341–360. 10.1042/EBC20170104 30030364 PMC6056715

[B103] Tsang S. H. Aycinena A. R. P. Sharma T. (2018). Mitochondrial disorder: kearns-sayre syndrome. *Adv. Exp. Med. Biol.* 1085 161–162. 10.1007/978-3-319-95046-4_30 30578503

[B104] Twig G. Elorza A. Molina A. J. Mohamed H. Wikstrom J. D. Walzer G. (2008). Fission and selective fusion govern mitochondrial segregation and elimination by autophagy. *EMBO J.* 27 433–446. 10.1038/sj.emboj.7601963 18200046 PMC2234339

[B105] van Loon B. Samson L. D. (2013). Alkyladenine DNA glycosylase (AAG) localizes to mitochondria and interacts with mitochondrial single-stranded binding protein (mtSSB). *DNA Repair (Amst.)* 12 177–187. 10.1016/j.dnarep.2012.11.009 23290262 PMC3998512

[B106] Vives-Bauza C. Zhou C. Huang Y. Cui M. de Vries R. L. Kim J. (2010). PINK1-dependent recruitment of Parkin to mitochondria in mitophagy. *Proc. Natl. Acad. Sci. U. S. A.* 107 378–383. 10.1073/pnas.0911187107 19966284 PMC2806779

[B107] Walker M. A. Lareau C. A. Ludwig L. S. Karaa A. Sankaran V. G. Regev A. (2020). Purifying selection against pathogenic mitochondrial DNA in human T cells. *N. Engl. J. Med.* 383 1556–1563. 10.1056/NEJMoa2001265 32786181 PMC7593775

[B108] Wallace D. C. (2005). A mitochondrial paradigm of metabolic and degenerative diseases, aging, and cancer: a dawn for evolutionary medicine. *Annu. Rev. Genet.* 39 359–407. 10.1146/annurev.genet.39.110304.095751 16285865 PMC2821041

[B109] Wallings R. Manzoni C. Bandopadhyay R. (2015). Cellular processes associated with LRRK2 function and dysfunction. *FEBS J.* 282 2806–2826. 10.1111/febs.13305 25899482 PMC4522467

[B110] Wang Y. Bogenhagen D. F. (2006). Human mitochondrial DNA nucleoids are linked to protein folding machinery and metabolic enzymes at the mitochondrial inner membrane. *J. Biol. Chem.* 281 25791–25802. 10.1074/jbc.M604501200 16825194

[B111] Waters L. S. Minesinger B. K. Wiltrout M. E. D’Souza S. Woodruff R. V. Walker G. C. (2009). Eukaryotic translesion polymerases and their roles and regulation in DNA damage tolerance. *Microbiol. Mol. Biol. Rev.* 73 134–154. 10.1128/MMBR.00034-08 19258535 PMC2650891

[B112] Wei W. Pagnamenta A. T. Gleadall N. Sanchis-Juan A. Stephens J. Broxholme J. (2020). Nuclear-mitochondrial DNA segments resemble paternally inherited mitochondrial DNA in humans. *Nat. Commun.* 11:1740. 10.1038/s41467-020-15336-3 32269217 PMC7142097

[B113] Wei Y. Chiang W. C. Sumpter R. Jr. Mishra P. Levine B. (2017). Prohibitin 2 is an inner mitochondrial membrane mitophagy receptor. *Cell* 168(1–2) 224–238.e10. 10.1016/j.cell.2016.11.042 28017329 PMC5235968

[B114] West A. P. Khoury-Hanold W. Staron M. Tal M. C. Pineda C. M. Lang S. M. (2015). Mitochondrial DNA stress primes the antiviral innate immune response. *Nature* 520 553–557. 10.1038/nature14156 25642965 PMC4409480

[B115] Wollert T. (2019). Autophagy. *Curr. Biol.* 29 R671–R677. 10.1016/j.cub.2019.06.014 31336079

[B116] Wonnapinij P. Chinnery P. F. Samuels D. C. (2008). The distribution of mitochondrial DNA heteroplasmy due to random genetic drift. *Am. J. Hum. Genet.* 83 582–593. 10.1016/j.ajhg.2008.10.007 18976726 PMC2668051

[B117] Wright W. D. Shah S. S. Heyer W. D. (2018). Homologous recombination and the repair of DNA double-strand breaks. *J Biol Chem* 293 10524–10535. 10.1074/jbc.TM118.000372 29599286 PMC6036207

[B118] Yakes F. M. Van Houten B. (1997). Mitochondrial DNA damage is more extensive and persists longer than nuclear DNA damage in human cells following oxidative stress. *Proc. Natl. Acad. Sci. U. S. A.* 94 514–519. 10.1073/pnas.94.2.514 9012815 PMC19544

[B119] Yamazaki T. Kirchmair A. Sato A. Buque A. Rybstein M. Petroni G. (2020). Mitochondrial DNA drives abscopal responses to radiation that are inhibited by autophagy. *Nat. Immunol.* 21 1160–1171. 10.1038/s41590-020-0751-0 32747819

[B120] Yang L. Long Q. Liu J. Tang H. Li Y. Bao F. (2015). Mitochondrial fusion provides an ‘initial metabolic complementation’ controlled by mtDNA. *Cell Mol. Life Sci.* 72 2585–2598. 10.1007/s00018-015-1863-9 25708700 PMC11113443

[B121] Yang W. Gao Y. (2018). Translesion and repair DNA polymerases: diverse structure and mechanism. *Annu. Rev. Biochem.* 87 239–261. 10.1146/annurev-biochem-062917-012405 29494238 PMC6098713

[B122] Yasukawa T. Kang D. (2018). An overview of mammalian mitochondrial DNA replication mechanisms. *J. Biochem.* 164 183–193. 10.1093/jb/mvy058 29931097 PMC6094444

[B123] Yoo S. M. Jung Y. K. (2018). A molecular approach to mitophagy and mitochondrial dynamics. *Mol. Cells* 41 18–26. 10.14348/molcells.2018.2277 29370689 PMC5792708

[B124] Youle R. J. van der Bliek A. M. (2012). Mitochondrial fission, fusion, and stress. *Science* 337 1062–1065. 10.1126/science.1219855 22936770 PMC4762028

[B125] Youn C. K. Kim H. B. Wu T. T. Park S. Cho S. I. Lee J. H. (2017). 53BP1 contributes to regulation of autophagic clearance of mitochondria. *Sci. Rep.* 7:45290. 10.1038/srep45290 28345606 PMC5366885

[B126] Yu W. Sun Y. Guo S. Lu B. (2011). The PINK1/Parkin pathway regulates mitochondrial dynamics and function in mammalian hippocampal and dopaminergic neurons. *Hum. Mol. Genet.* 20 3227–3240. 10.1093/hmg/ddr235 21613270 PMC3140825

[B127] Zheng J. Croteau D. L. Bohr V. A. Akbari M. (2019). Diminished OPA1 expression and impaired mitochondrial morphology and homeostasis in Aprataxin-deficient cells. *Nucleic Acids Res.* 47 4086–4110. 10.1093/nar/gkz083 30986824 PMC6486572

[B128] Zong W. X. Rabinowitz J. D. White E. (2016). Mitochondria and cancer. *Mol. Cell* 61 667–676. 10.1016/j.molcel.2016.02.011 26942671 PMC4779192

[B129] Zorov D. B. Popkov V. A. Zorova L. D. Vorobjev I. A. Pevzner I. B. Silachev D. N. (2017). Mitochondrial aging: is there a mitochondrial clock? *J. Gerontol. A Biol. Sci. Med. Sci.* 72 1171–1179. 10.1093/gerona/glw184 27927758

